# Effect of home-based online training and activity feedback on oxygen uptake in patients after surgical cancer therapy: a randomized controlled trial

**DOI:** 10.1186/s12916-023-03010-6

**Published:** 2023-08-08

**Authors:** Roberto Falz, Christian Bischoff, René Thieme, Uwe Tegtbur, Peter Hillemanns, Jens-Uwe Stolzenburg, Bahriye Aktas, Ulrich Bork, Jürgen Weitz, Johannes Lässing, Christian Leps, Johannes Voß, Florian Lordick, Antina Schulze, Ines Gockel, Martin Busse

**Affiliations:** 1 Institute of Sport Medicine and Prevention, University Leipzig, Rosa-Luxemburg-Str. 30, Leipzig, 04103 Germany; 2 Department of Visceral, Transplant, Thoracic and Vascular Surgery, University Hospital Leipzig, Leipzig, Germany; 3 Institute of Sport Medicine, Hannover Medical School, Hannover, Germany; 4 Department of Gynecology and Obstetrics, Hannover Medical School, Hannover, Germany; 5 Department of Urology, University Hospital Leipzig, Leipzig, Germany; 6 Department of Gynaecology, University Hospital Leipzig, Leipzig, Germany; 7 Department of Visceral-, Thoracic and Vascular Surgery, University Hospital Carl Gustav Carus, Technische Universität Dresden, Dresden, Germany; 8 National Center for Tumor Diseases (NCT), Partner Site Dresden, Dresden, Germany; 9 Institute of Exercise Science & Sports Medicine, Martin Luther University Halle-Wittenberg, Halle, Germany; 10 Department of Oncology, Gastroenterology, Hepatology, Pulmonology and Infectious Diseases, University Hospital Leipzig, Leipzig, Germany; 11 University Cancer Center Leipzig, University Hospital Leipzig, Leipzig, Germany

**Keywords:** Cardiac output, Activity feedback, Adherence, Body composition, Strength-endurance training, Home-based exercise

## Abstract

**Background:**

Exercise training is beneficial in enhancing physical function and quality of life in cancer patients. Its comprehensive implementation remains challenging, and underlying cardiopulmonary adaptations are poorly investigated. This randomized controlled trial examines the implementation and effects of home-based online training on cardiopulmonary variables and physical activity.

**Methods:**

Of screened post-surgical patients with breast, prostate, or colorectal cancer, 148 were randomly assigned (1:1) to an intervention (2 × 30 min/week of strength-endurance training using video presentations) and a control group. All patients received activity feedback during the 6-month intervention period. Primary endpoint was change in oxygen uptake after 6 months. Secondary endpoints included changes in cardiac output, rate pressure product, quality of life (EORTC QoL-C30), C-reactive protein, and activity behavior.

**Results:**

One hundred twenty-two patients (62 intervention and 60 control group) completed the study period. Change in oxygen uptake between intervention and control patients was 1.8 vs. 0.66 ml/kg/min (estimated difference after 6 months: 1.24; 95% CI 0.23 to 2.55; *p* = 0.017). Rate pressure product was reduced in IG (estimated difference after 6 months: − 1079; 95% CI − 2157 to − 1; *p* = 0.05). Physical activity per week was not different in IG and CG. There were no significant interaction effects in body composition, cardiac output, C-reactive protein, or quality of life.

**Conclusions:**

Home-based online training among post-surgery cancer patients revealed an increase of oxygen uptake and a decrease of myocardial workload during exercise. The implementation of area-wide home-based training and activity feedback as an integral component in cancer care and studies investigating long-term effects are needed.

**Trial registration:**

DRKS-ID: DRKS00020499; Registered 17 March 2020.

**Supplementary Information:**

The online version contains supplementary material available at 10.1186/s12916-023-03010-6.

## Background

Breast, prostate, and colorectal cancer rank among the most commonly diagnosed cancers and leading cause of cancer-related deaths worldwide and in Europe [1, 2]. The burden of cancer disease remains a significant health issue resulting from the cancer symptoms, its chemotherapeutic and surgical treatment, and related comorbidities such as developing heart disease [3]. Cancer is also associated with aging [4] and cardiorespiratory fitness reveals a strong inverse correlation with cancer mortality [5].

The role of exercise in cancer has attracted significant research interest over the past decades [6, 7]. There is strong evidence for improving cancer-related health outcomes via exercise in terms of anxiety, depressive symptoms, fatigue, health-related quality of life, lymphedema, and physical function and that exercise training is generally safe for cancer survivors [8]. Overall, physical activity is effective for the prevention of several cancer entities [7, 9–13]. Exercise training or physical activity after a cancer diagnosis is beneficial for overall survival or preventing a recurrence of breast (app. 20–30%), prostate (app. 5–30%), and colorectal cancer (app. 20–30%) [7, 14–16]. Post-diagnosis exercise training seems to exert a stronger effect on cancer outcomes in comparison to pre-diagnosis exercise [7]. There is strong evidence that cardiorespiratory fitness or changes in cardiorespiratory fitness are inverse and independently associated with all-cause mortality risk [17–19]. Potentially beneficial biological mechanisms of exercise on cancer are the modulation of insulin/glucose metabolism and thus reducing obesity, inflammation, and oxidative stress, which reduces tumor growth, the activation of tumor suppressor genes, and an increase in apoptosis in tumor tissue [20–26]. Exercise also supports the mechanisms of chemotherapeutic agents [20, 27].

The current physical activity guidelines recommend 150 to 300 min per week of moderate (3 to 5.9 METs) or an equivalent amount of vigorous intensity aerobic activity of 75 to 150 min per week (< 6 METs) [28]. However, in patients undergoing chemotherapy, chemo radiotherapy, or cancer surgery, these recommendations should be adapted to the patients’ individual performance level. The type, intensity, and amount of training must therefore be individually set according to the patient’s own performance and postsurgical condition. The objectification of physical activity is essential, when assessing dose–response relationships between exercise interventions and their effects in cancer patients [29], but actual physical activity is usually self-reported and thus not reliably objectifiable [30]. Furthermore, essential factors that help patients maintain their adherence to a training program include considering their individual capacity, giving them motivation-enhancing activity feedback, and bidirectional communication [31]. Telemedicine-based exercise interventions in cancer patients enable measured activity tracking, communicating with and among patients, and have revealed (long-term) improvements on physical activity, self-management, and functional capacity [32–35]. Nevertheless, the effects of home-based exercise interventions on physical capacity reported to date were small [30], the induced cardiopulmonary adaptations have not yet been fully elucidated, and home-based exercise seems a safe and feasible intervention strategy for patients with cancer [6, 8, 36] although there is little published evidence for assessment to date [36, 37]. Lastly, despite the positive evidence of physical training on cancer outcomes, there has to date been no systematic implementation or large randomized controlled trails of individually-adapted exercise training involving quantitative activity feedback after cancer surgery.

Given the uncertainty of the role of home-based online training and activity feedback in post-surgery cancer patients, the aim of this trial was to test whether online-supported training (intervention) and online activity feedback (intervention and control group) result in different changes in oxygen uptake and cardiopulmonary exercise parameters, quality of life, and activity behavior during and after 6 months.

## Methods

### Study design, ethics approval, and patients

**Fig. 1:**
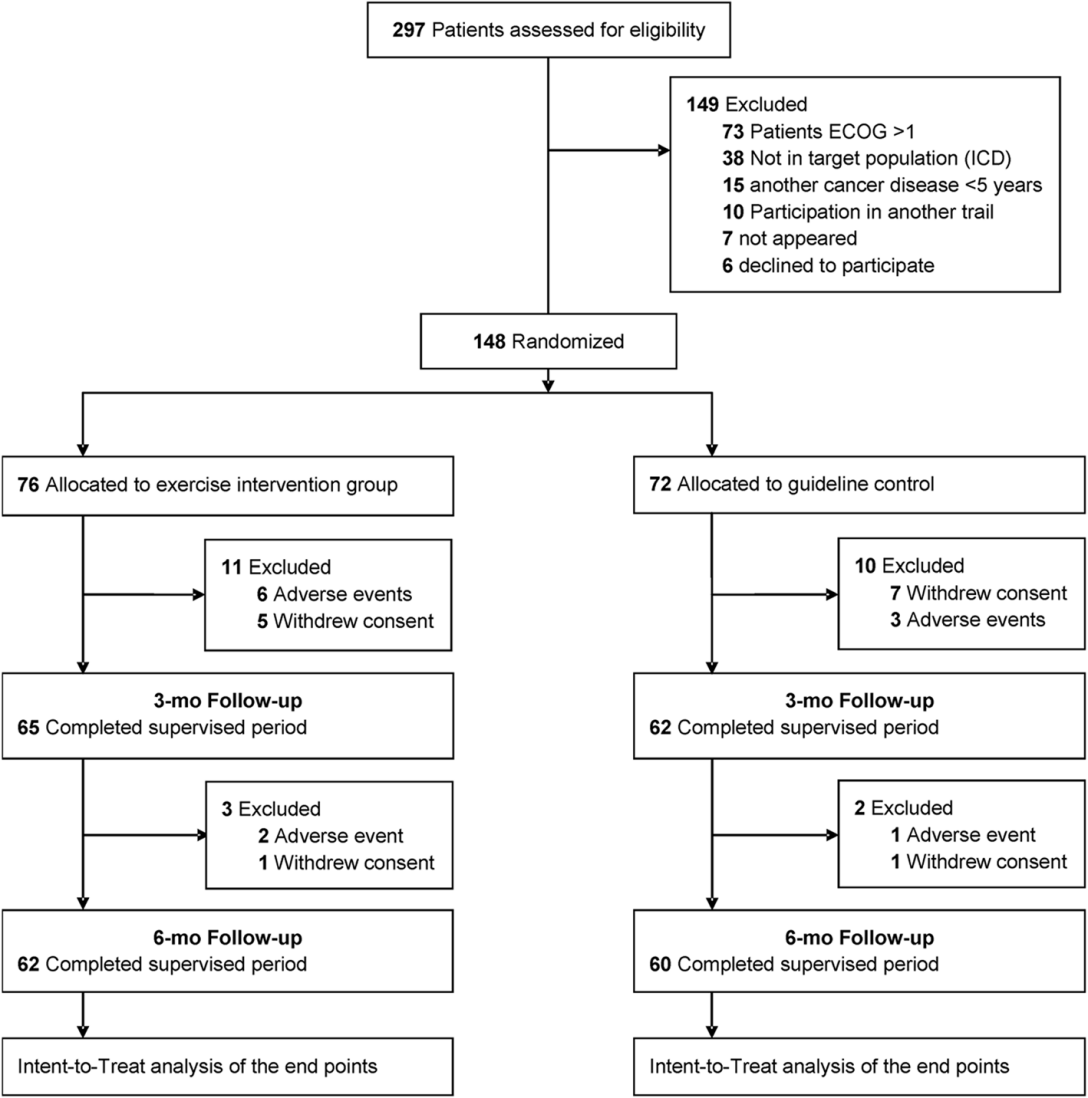
CONSORT flow diagram

CRBP-TS (ColoRectal, Breast, and Prostate Cancer—Telemonitoring and Self-management) was a randomized, multicenter trial involving an intervention (IG) and control group (CG) that assessed supervised and home-based post-surgery online training to strengthen physical performance and patient empowerment via automated activity feedback information after cancer surgery. A detailed description of the study design has been published [38]. The study was approved by the Ethics Committee of the Medical Faculty, University of Leipzig (reference number 056/20-ek), and at all participating sites. The patients were screened, informed, and enrolled at University Hospitals in Dresden, Hannover, and Leipzig. All participants provided written informed consent. Cancer patients with International Classification of Diseases codes C18/19/20 (colorectal cancer), C50 (breast cancer), and C61 (prostate cancer) who underwent curative (R0) surgery at stages T1N0M0 to T3N3M0 (Tumor, Nodes, Metastases; including M1 with achieved R0 resection); ECOG (Eastern Cooperative Oncology Group) < 1; and aged between 18 to 75 years were eligible to participate in this trial. In total, 148 patients were recruited and randomized (Fig. 1 and Table 1).

**Table 1:** Demographic and clinical characteristics at baseline

	Intervention group (*n* = 76)	Control group (*n* = 72)
Age (years)	54.4 ± 11	54.6 ± 12
Sex		
Female (%)	45 (59)	43 (60)
Male (%)	31 (41)	29 (40)
Height (cm)	172 ± 8	171 ± 11
Body composition		
Weight (kg)	78.8 ± 15.3	74.9 ± 15.3
Fat mass (kg)	24.6 ± 10.3	21.3 ± 8.4
Lean body mass (kg)	54.2 ± 11.1	53.6 ± 10.6
Body mass index (kg/m^2^)	26.9 ± 4.5	25.1 ± 4.7
Waist-to-hip ratio	0.90 ± 0.2	0.88 ± 0.1
Cancer entity no. (%)		
Colorectal cancer	10 (13)	9 (12)
Breast cancer	43 (57)	41 (57)
Prostate cancer	23 (30)	22 (31)
Dropouts no. (%)	14 (18)	12 (17)
SAE nos. (hospitalizations)	11	7
Comorbidities, no (%)		
Diabetes type 2	4	2
Hypertension	23	17
Adiposity (BMI > 30)	16	11
Cardiovascular diseases	2	3
Hypothyroidism	13	15
Asthma	2	2
Arthritis	9	3
Cancer medication		
Estrogen receptor modulator	9	16
Monoclonal antibody	2	1
Aromatase inhibitors	8	4
Chemotherapy medication	3	2
Metabolic status		
CRP level (mg/l)*	1.5 (0.7; 2.5)	1.0 (0.6; 1.9)
HbA_1c_ (%)	5.4 ± 0.4	5.4 ± 0.7
Albumin level (g/l)	45.0 ± 2.8	45.3 ± 2.5
Hemoglobin level (mmol/l)	8.5 ± 0.85	8.4 ± 0.8
Ferritin (ng/l)	150 ± 189	180 ± 199

Values are presented as the means and standard deviation or as the median* and 25th and 75th percentiles*

*Abbreviations*: *CRP* C-reactive protein, *HbA*_*1c*_ *g*lycosylated hemoglobin

### Randomization

An online-based system was used to assign patients in a 1:1 allocation to IG or CG. Randomization was stratified by study site and cancer entity using block sizes of two and four blocks.

### Intervention and CRBP-TS application

Home-based and body-weight online training consisted of entity-specific, individually performance-adapted, and heart rate-limited strength-endurance training using video presentations. Individual entry and follow-up levels based on patients’ individual physical performance level were used. The training was scheduled in accordance with exercise guidelines [8, 28] for two (at least) or preferably three times or more per week for 30 min per session (5-min warm-up; four rounds with five different body-weight upper and lower body exercises with 40 s loading and 20 s recovery period per set; 5-min cool down). The strength endurance exercises mainly done with the patient’s own body weight included for example stepping exercises, squats, rowing, upper body push and pull exercises, jumps, and core exercises. For the included entities, partially adapted training videos were designed (i.e., breast cancer patients avoided shoulder exercises, colorectal cancer patients avoided exercises in prone position, and prostate cancer patients avoided jumping exercises). The target training intensity was determined by the perceived exertion (target 5–8; CR10 scale) [39–41], and adjustable after each training month’s period, because strength endurance training (interval exercise with predominantly peripheral fatigue) cannot be adequately controlled by heart rate zones. Nevertheless, a heart rate sensor was used to document exercise intensity during exercise sessions and to enable immediate feedback to patients. For forensic reasons, an individual maximum heart rate (75% heart rate max or symptom-limited heart rate) was defined using a cardiopulmonary exercise test) at baseline and was individually adjustable during a repeated cardiopulmonary exercise test after 3 months (study visit 2).

At baseline, all patients were given a wearable (Vivo active 4; Garmin, Olathe, Kansas, US) for activity tracking (steps per day, active minutes per day with an intensity > 3 MET) connected to a tablet (Lenovo Tab M10 TB-X606X; Lenovo, Hongkong, China) via Bluetooth with the CRBP-TS application. The wearable unit was to be worn 24 h a day during the entire study period. An automatic data transfer from patient to an electronic patient file (case report form) was provided via internet access (LTE, Deutsche Telekom AG, Germany) in the tablet device. The CRBP-TS application was meant to be regularly used by the patients to visualize the training video presentations, to record their heart rate (chest belt) and to receive their heart-rate feedback during the training sessions, to complete questionnaires, and to receive physical activity feedback (steps per day, activity time in minutes > 3 MET per week) during the intervention period. Structured information on general health improvement, disease prevention, and lifestyle changes (diet, training, and self-perception) was also provided by the study team (physician, sports scientist, and a study nurse) via the CRBP-TS application. In case of unscheduled exercise breaks, patients were encouraged via short message service as reminders to increase their adherence. CG patients received general information about lifestyle changes and physical activity according to guidelines and, as described in the IG, the wearable and a tablet with the CRBP-TS application to enable the receipt of activity feedback information (without video presentations).

### Clinical assessments

All patients were assessed at baseline and 3 and 6 months (study visit 2 and 3) after randomization. The CRBP-TS application measured and evaluated the daily activity of patients in the IG and CG. All study subjects underwent clinical examinations according to standard operating procedures; this included medical history, anthropometry (BIACORPUS RX 4004 M, MEDI CAL HealthCare GmbH, Germany), flow-mediated dilation, blood analysis (clinical chemistry; tumor and inflammation marker panel) cardiopulmonary exercise testing, and completing the European Organization for Research and Treatment of Cancer Quality of Life Questionnaire (EORTC QoLQ-C30). Subjects also underwent cardiopulmonary exercise testing to determine primary and secondary end points (custo, BT300 electrocardiogram, custo GmbH, Germany; PhysioFlow impedance cardiography, Manatec Biomedical, France; Dynostics ergo-spirometry, Sicada GmbH, Germany) on an electronically braked semi-recumbent ergometer. The initial load was 30 Watts with 10 Watts/min increments until subjective or objective exhaustion or the occurrence of termination criteria [42].

Staff members conducting the evaluations were not blinded to treatment groups. Cardiopulmonary exercise capacity was tested according to current recommendations [42] and analyzed in blinded manner at the study core laboratory in Leipzig. Maximum oxygen uptake (V̇O_2max_) was defined as the highest 30-s average within the last minute of exercise. Blood parameters were analyzed at the central core laboratory (Institute of Laboratory Medicine, Clinical Chemistry and Molecular Diagnostics, University Hospital Leipzig, Leipzig, Germany).

### Outcomes

Our primary end point was the change in V̇O_2max_ after 6 months (study visit 3). Secondary end points included changes from baseline to 6 months for cardiopulmonary exercise testing parameters (cardiac output [CO], rate pressure product [RPP], and peak power output), anthropometric parameters (body mass index [BMI], body cell mass [BCM]), C-reactive protein (CRP), and the quality of life (EORTC QoL-C30). Activity parameters (steps per day, active minutes > 3 MET per week) were recorded during the entire study period in IG and CG. The additional secondary end points of changes in flow-mediated dilatation, blood parameters (inflammation panel, tumor makers, liquid biopsies, and metabolic markers), questionnaires (Patient Health Questionnaire-2; Depression Anxiety Stress Scale; Fatigue Severity Scale and Oral Health Impact Profile), and other anthropometric parameters (fat mass, lean body mass) from baseline to 6 months are not reported here. Serious adverse events (SAE) were documented and categorized at each study site and then evaluated. To identify SAEs, participants were asked to self-report any health problems during the study period and were questioned about events by the site investigator at each study visit. Selection criteria were death, life-threatening, hospitalization, disability, or permanent damage. SAE reports were assessed according to GCP ICH by the study site principal investigator (temporal or exercise association with training; alternative relationship, e.g., accident or new illness) and reported to the head of clinical trial.

### Statistics

The trial protocol defined 13% change in V̇O_2max_ in the IG compared to the CG as clinical or substantial importance [43, 44]. By an assumed mean difference of 3.6 ml/kg/min between home-based online training and the CG, 80% power and an alpha of 0.05, a sample containing 40 patients per group was needed. Considering probable dropouts and a moderate number of missing values, we aimed to include 100 patients (50 in IG and CG) in this study. To enable a subgroup analysis per entity (breast, prostate and colorectal cancer), we needed to enroll 300 patients.

Data were analyzed using IBM SPSS Statistics (Version 29; IBM, Armonk, New York, USA) and displayed using GraphPad Prism (Version 9; GraphPad Software Inc., California, USA). The Shapiro–Wilk test was used to analyze the sampling distribution. The evaluation was conducted on an intention-to-treat basis, and all randomized participants were included. Available data on participants with missing data were included under the “missing at random” assumption. Per-protocol analyses were conducted, including only participants in the IG who completed all study visits and who had engaged in at least 1.5 training sessions per week.

To evaluate the primary and secondary end points, we applied mixed-effects models with repeated measurement structure (estimated using restricted maximum likelihood). In this model, the measured values (measured at baseline, 3-month, and 6-month follow-up) were treated as the dependent variable. As fixed effects, we have included the randomization arm and categorical time covariate in the model. Interactions were modeled for group and time (categorical). As random effect(s), we had an intercept for subjects. Within the mixed models, we estimated 95% confidence intervals (CI) and *p*-values for contrasts between groups for the 3- and 6-month periods. In a sensitivity analysis, we included only those patients with complete paired baseline and 6-month follow-ups for time difference within groups (paired *t* test for dependent samples). Sphericity was checked using Mauchly’s *W* test, and Greenhouse–Geisser correction was applied when necessary. All analyses were two-sided, and the level of significance was *p* = 0.05.

## Results

The first patient was enrolled in July 2020, with the last patient completed the study in June 2022. After screening 297 patients, we could enroll 148 patients in the study (Fig. 1). A total of 149 patients therefore had to be excluded for the following reasons: 73 for having an Eastern Co-operative Oncology Group (ECOG) status of > 1, 38 screened patients because of an ICD code or TNM status discrepancy, 15 because of additional tumor disease within the past 5 years, 13 patients did not appear for baseline or withdrew informed consent, and 10 because of participating in another clinical trial at the time of screening. No participants were excluded from our intent-to-treat analysis. Twenty-six patients dropped out of the study during the 6-month study period. Dropout reasons included new medical complaints or diagnoses, lack of interest, problems using the app/technology, and other reasons (total no-shows). Baseline patient demographics and clinical data (mean age, 54 years; 88 women [59%]; mean BMI, 26.0) are shown in Table 1.

### Primary outcome

**Fig. 2:**
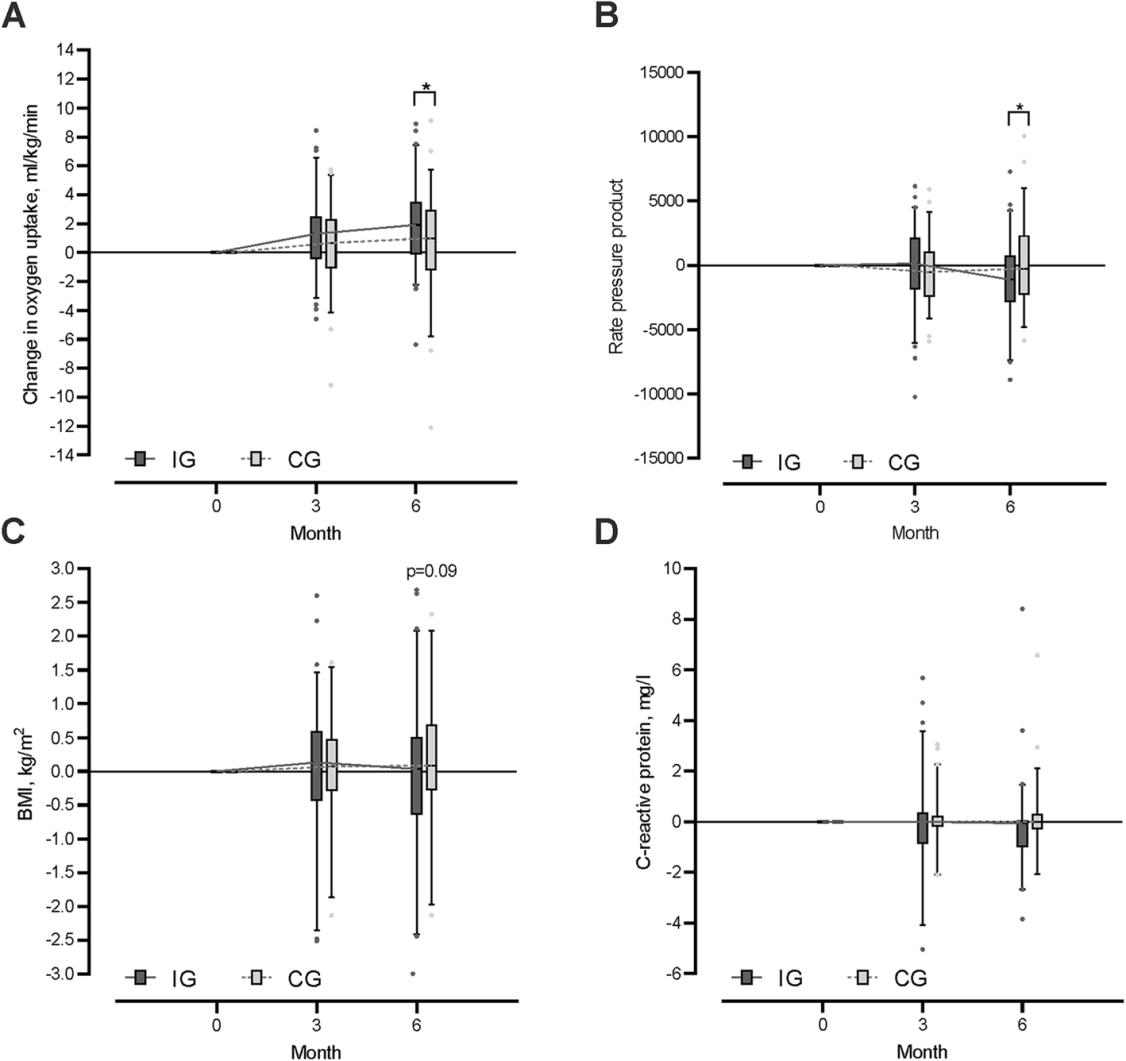
Change in oxygen uptake (**A**), rate blood pressure product (**B**), body mass index (**C**), and C-reactive protein concentration (**D**) at 3 and 6 months in IG and CG. Changes are calculated from baseline to 3 and 6 months of intervention within each group (presented as boxes and whiskers [median, quartiles, 5 to 95 percentiles]. *Significant difference (*p* < 0.05) between change in IG and CG

The mixed effect model of repeated measurement of maximal oxygen uptake showed a significant time effect (*p* < 0.001), a *p*-value of 0.056 for interaction effect and no group effect (Table 2). After the 6-month intervention, the estimated change in V̇O_2max_ differed significantly between groups 1.24 [95% CI: 0.23 to 2.25: *p* = 0.017] mL/kg/min. The IG’s increase in V̇O_2max_ from baseline to 6 months was significant (mean [SD] for IG: 1.82 [2.7] mL/kg/min), unlike the CG’s (mean [SD] for CG: 0.66 [3.5] mL/kg/min). Figure 2 displays the change in V̇O_2max_ across the study visits at 3 and 6 months. The estimated change at the 3-month time point showed no significant group difference. Subgroup analysis per entity for the change in oxygen uptake is reported in Table S1 (additional file 1) and 3-month visit data are presented in Table S2 (additional file 1).

**Table 2:** Primary and secondary end points after 6 months (intent to treat analysis)

	Mean (SD) [sample size]							Time effect^c^	Group effect^c^	Inter-action effect^c^
	**Intervention group**			**Control group**			Difference^b^ 6 months IG vs. CG (95% CI)	*p*	*p*	*p*
	**Pre**	**6 mo**	**Diff**^**a**^	**Pre**	**6 mo**	**Diff**^**a**^				
VO_2max_, ml/kg/min	26.2 (6.0) [74]	28.1 (6.7) [61]	1.82* (2.71) [61]	28.1 (6.3) [71]	29.3 (7.1) [58]	0.66 (3.50) [58]	**1.24**^#^ (0.23 to 2.25)	**< 0.001**	0.25	0.056
VO_2max_, ml/min	2045 (454) [74]	2197 (528) [61]	126* (239) [61]	2065 (501) [71]	2149 (556) [58]	55 (276) [58]	76 (− 7 to 158)	**< 0.001**	0.81	0.17
Peak power output, watt	132 (33.3) [74]	145 (38.9) [61]	10.2* (16.2) [61]	135 (34.1) [71]	145 (36.6) [58]	6.3 (18.7) [58]	3.8 (− 1.9 to 9.5)	**< 0.001**	0.66	0.28
Rate pressure product^d^	26468 (5539) [73]	25258 (5677) [61]	− 1109* (3164) [61]	26715 (5099) [71]	26382 (4641) [58]	94.2 (3249) [58]	**− 1079**^#^ (− 2157 to − 1)	0.16	0,56	**0.04**
Cardiac output max, l/min	16.7 (3.3) [61]	17.7 (3.4) [55]	0.59 (2.5) [46]	17.7 (2.8) [59]	17.9 (3.5) [51]	0.17 (2.7) [43]	0.52 (− 0.4 to 1.5)	0.28	0.25	0.40
CRP, mg/l	3.2 (7.6) [76]	1.7 (1.8) [62]	− 0.7 (4.3) [62]	3.4 (14.5) [71]	1.6 (1.6) [59]	− 2.1 (16.0) [59]	0.4 (− 3.4 to 4.2)	0.22	0.85	0.66
Body cell mass, kg	30.1 (5.8) [74]	31.4 (6.6) [63]	1.0* (2.2) [63]	29.9 (6.2) [69]	30.5 (6.7) [58]	0.7* (1.7) [58]	0.3 (− 0.3 to 0.9)	**< 0.001**	0.75	0.59
BMI, kg/m^2^	26.6 (4.7) [74]	26.9 (4.5) [63]	− 0.1 (1.1) [63]	25.3 (4.4) [71]	25.1 (4.1) [60]	0.2 (0.9) [60]	− 0.3 (− 0.6 to 0.03)	0.81	0.11	0.16
EORTC QLQ-C30; global score	62.4 (21.6) [68]	67.4 (25.4) [54]	4.6 (33.4) [49]	60.1 (22.9) [65]	69.3 (18.8) [54]	13.1* (31.5) [50]	− 4.5 (− 15 to 6.3)	**0.01**	0.86	0.61
Activity per week^e^ (> 3 MET), min	145 (160) [74]	124 (141) [68]	− 31 (129) [67]	170 (152) [69]	114 (111) [60]	− 48* (116) [58]	24.4 (− 14 to 63)	**< 0.001**	0.55	0.44
App use per week^e^ numbers	26.2 (7.7) [75]	20.8 (9.4) [70]	− 5.9* (9.2) [69]	21.2 (6.6) [71]	16.6 (7.1) [62]	− 5.0* (7.0) [62]	− 0.8 (− 3.1 to 1.6)	**< 0.001**	**< 0.001**	0.74

*Abbreviations*: *pre* baseline, *mo* months, *diff* difference, *VO*_*2max*_ maximum oxygen uptake, *MET* metabolic equivalent of tasks, *BMI* body mass index, *EORTC QLQ* European Organization for Research and Treatment of Cancer Quality of Life questionnaire

^*^Significant difference (*p* < 0.05; within groups)

^#^Significant difference (*p* < 0.05; between groups)

^a^Sensitive analysis: results of the complete case analysis considering all available data

^b^Estimates of differences between group changes

^c^Main effects of mixed-effects models

^d^For the same absolute power output

^e^Pre = week 1 to week 8 and 6 mo = week 17 to week 25

### Secondary outcomes

**Fig. 3:**
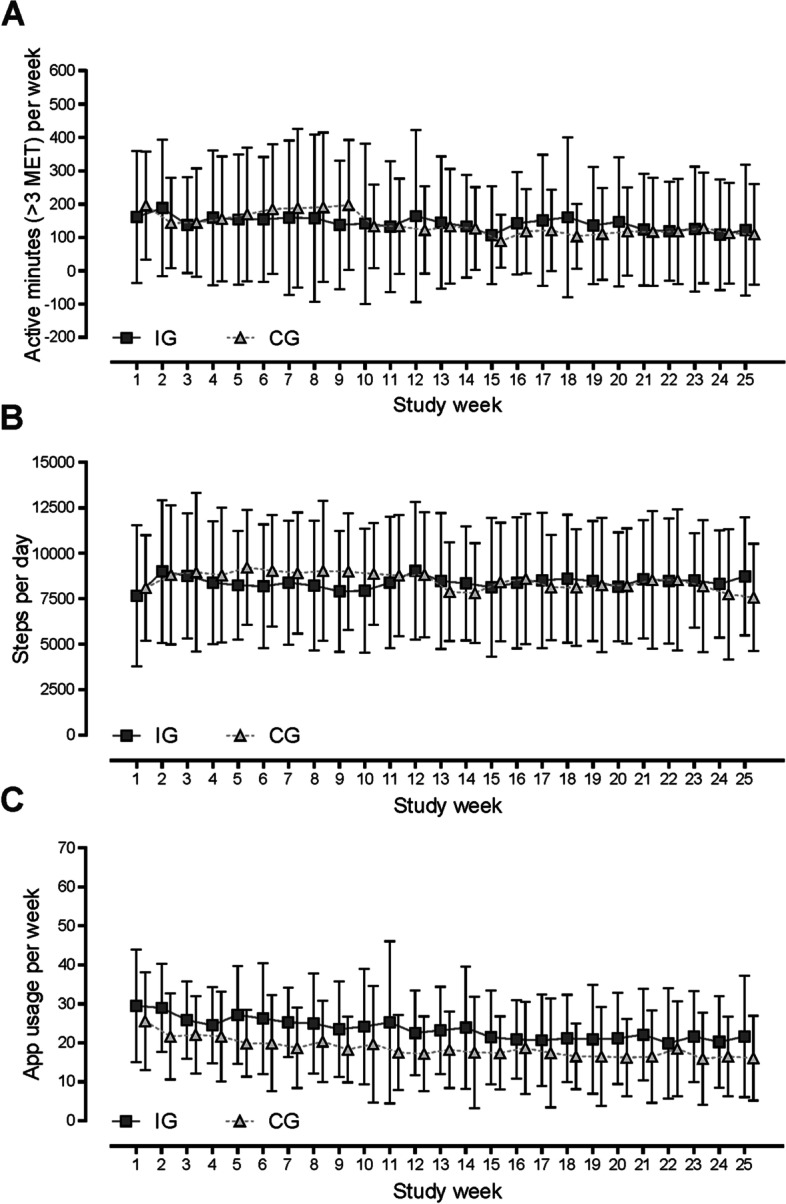
Active minutes above 3 MET per week (**A**), steps per day (**B**), and number of app uses per week (**C**) during the study period in IG and CG (presented as mean and standard deviation)

The increase in *peak power output* after 6 months showed a significant time effect in the mixed model (*p* < 0.001) and within groups without significant differences between IG and CG (3.8 W [95% CI, − 1.9 to 9.5]). However, after 6 months, the change in the *rate pressure product (RPP)* was significantly lower in the IG than the CG (− 1079 [95% CI, − 2157 to − 1]; Table 2, Fig. 2B) and showed an interaction effect in the mixed model analysis (*p* = 0.04). Nonsignificant increases during the intervention period in *cardiac output* (CO) were observed within the IG and in the global mixed model analysis with no group difference (0.4 l/min [95% CI, − 4.2 to 3.4]; Table 2). Change in *BMI* after 6 months was not significant and showed no group differences (Table 2). The difference between the change in IG und CG was 0.3 [95% CI, − 0.6 to 0.03]. There was no significant difference in either the change in *C-reactive protein* (CRP) within groups over time or between groups (Table 2, Fig. 2D). Nevertheless, *body cell mass* (BCM) rose significantly in both groups, although no group differences were evident (Table 2). The change in *quality of life* (QoL) revealed a significant increase over time in the CG, but this did not differ significantly between groups (Table 2). The activity data (activity in minutes per week > 3 MET) revealed no group difference between IG and CG patients in the (Table 2). The CG showed a decrease in *activity behavior* over time (− 48 min per week > 3 MET). There was no interaction effect. Patients randomized to IG demonstrated throughout the study period (week 1 to week 25) 128 (SD ± 135) minutes of total activity > 3 MET and 29 (SD ± 32) minutes activity with intensity exceeding 6 MET per week. The CG revealed a mean of 142 (SD ± 122) minutes total activity > 3 MET and 31 (SD ± 37) minutes activity > 6 MET per week during the 6-month study period (week 1 to week 25). App usage in both groups decreased significantly during the study period and differed between groups but showed no interaction effect (Table 2). Figure 3 illustrates the course of activity (steps per day; active minutes > 3 MET) and app usage per week during the entire study period in IG and CG.

### Adherence and per-protocol analysis

One hundred twenty-two patients completed the 6-month study period (62 in IG, 60 in CG). *Dropouts* (IG: *n* = 14; CG: *n* = 12) were mainly for clinical reasons (*n* = 9; hospitalizations or worsening health) or consent withdrawal (*n* = 17; motivational problems [*n* = 6], difficulty handling the devices [*n* = 6], other reasons [*n* = 5]). Patients randomized to the IG participated in a mean of 2.1 (SD ± 1.1) training sessions per week in the intervention period.

#### Adherence

Of those patients who completed the 6-month training intervention, 46 (74.2%) performed at least 1.5 training sessions per week (mean 2.7; SD ± 1.0) and 164 (SD ± 152) minutes total activity > 3 MET per week across the entire study period (week 1 to week 25). Thirty-five (56.4%) patients in IG even exercised more than 2 sessions per week (mean 3.0; SD ± 0.9) and were active 172 (SD ± 164) minutes per week (week 1 to week 25). Reductions in adherence to less than 1.5 of scheduled exercise sessions were mainly due to clinical factors (*n* = 6), personal (*n* = 2), and motivational problems (*n* = 8).

Results of the per-protocol analysis were similar to the main results of the trial (Additional file 1: Table S3).

### Adverse events

We documented 18 adverse events in 16 patients (11%) classified as serious adverse events (SAE) that were unrelated to the exercise intervention (no temporal relation to training, other cause such as accident, new disease diagnosis or scheduled surgery; IG 10 [13%] patients; CG 6 patients [8%]; chi-square *p* = 0.35). Hospitalization or disease progression was the cause for being classified as an SAE in all these cases. Reasons were scar hernia (*n* = 5; without timely relation to the training), metastases or recurrence (*n* = 3), accidents involving a trauma to the passive musculoskeletal system (*n* = 3), additional carcinoma (*n* = 2), stoma relocation (*n* = 2), pulmonary embolism (*n* = 1), peritonitis (*n* = 1), and urethral stenosis (*n* = 1).

## Discussion

In postoperative cancer patients, we observed that home-based strength-endurance online training entailing the provision of measured activity feedback information yielded a significant increase in V̇O_2max_ during the intervention period in the IG and compared to the CG, while other parameters were unaffected (CRP, BMI, QoL) (those had been in the reference range at baseline). Moreover, the IG revealed the a priori defined difference of 3.5 ml/kg/min at none of the study time points. Nevertheless, we were able to demonstrate an area-wide home-based online training and a significant decrease in rate pressure product during the exercise intervention in post-surgery cancer patients. Activity feedback seemed to induce in general a positive lifestyle modification toward physical activity in our CG because the recorded activity per week resembled the IGs.

The 1.24 ml/kg/min difference in V̇O_2max_ we observed between home-based online training and control patients of resembles such evidence from other studies (post-surgery supervised training, not home-based), reporting a range of 0.4 to 1.8 ml/kg/min [44–47]. However, our study results failed to confirm the findings of two smaller studies, which reported a difference of 4.0 ml/kg/min in postmenopausal women [48], or a reduction in male patients (*n* = 21) with colorectal cancer of − 1.7 [49] in comparison to the change in control patients. So far, home-based exercise or telerehabilitation interventions in cancer patients has demonstrated only minor to moderate effects on functional capacity, measured via the 6-min walking distance or oxygen uptake [50, 51]. Nevertheless, minor changes in cardiorespiratory performance result in an inverse and clinically relevant change in mortality risk and contribute to improved health [18]. *Peak power output* rose in both groups without interaction effects but is, nevertheless, in line with the change in V̇O_2max_. Cardiac dysfunction in cancer and caused by cancer-related-therapies is a frequent side effect requiring adequate diagnosis and interventional strategies [52, 53]. To the best of our knowledge, our randomized controlled study is the first to have measured maximum *cardiac output* (CO) during exercise testing before and after long-term exercise interventions in post-surgery cancer patients. So far, exercise data on CO in cancer patients has only been collected during acute exercise and under resting conditions [54–56]. Evidence from healthy subjects suggests that exercise training, particularly interval training, helps improve the pumping function [57] and seems to be significantly enhanced by enlarging the blood volume [58, 59]. However, the change in CO we observed was not significant and suggests home-based online training’s limited effect on cardiac pump function.

In contrast, as did two previous exercise trials in cancer patients [60, 61], the present study demonstrates that the improvement in exercise capacity after 6 months was associated with a reduction in the *rate pressure product* (RPP) as a reliable index of myocardial oxygen demand. The RPP decreased significantly further in the IG than the CG. These results highlight that despite equal physical activity in IG and CG and unchanged cardiac output, home-based online training (strength-endurance) contributed to the improvement we observed in V̇O_2_.

In our study, *QoL* improved in both groups (no within-change in IG, significant within-change in CG), but there was no apparent interaction effect. The study by Saarto et al. (2012) also assessed QoL using EORTC-QLQ C30 to determine changes in QoL during exercise interventions in breast cancer survivors, and supports our findings. However, in addition to improvements in physical performance through the exercise interventions, several meta-analyses have demonstrated small to moderate effects on QoL in cancer survivors [62–64]. Supervised training exerts a stronger effect than home-based training and may have contributed to our results [8]. The activity feedback in the CG—as in the IG—seems to be a significant factor stimulating motivation in our control patients. Nevertheless, the improvements in quality of life (EORTC-QoL C30, global score) we noted originating from the interventions resemble those reported in a recent meta-analysis that cited a mean increase of 4.4 [65] and were related to the change in physical fitness (V̇O_2max_).

The *physical activity time* of intervention and control patients involving an intensity exceeding 3 METs was measured during the entire study period while they were wearing their wrist-worn wearable, but this measure revealed no group differences. However, our CG revealed a significant reduction in activity time per week from baseline to their 6-month visit. Wrist-worn wearables enabling activity tracking are very accurate at monitoring the heart rate and numbers of steps [66, 67]; however, they can easily underestimate energy expenditure at high intensities and when activity involves less wrist motion [68, 69]. Our applied body-weight online training involves low arm movement amplitudes and frequencies, thus might have resulted in an underestimation of the IG’s physical activity. The changes in V̇O_2max_ and RPP via the same activity behavior of IG compared with the CG indicate a possible missing recording of activity during the training sessions. However, it seems also likely that home-based body-weight training is more effective in inducing performance-enhancing adaptations at the same overall activity level. Overall, distance-based physical activity interventions revealed only minor effects on activity behavior [30]. However, most of such evidence originates from self-reported physical activity, which compromises the comparability of studies associated with the subjective aspect of recoding such activity [8, 30]. Nevertheless, the effects that we have demonstrated, albeit small, are evidence that online or home-based exercise programs are a potential tool to address functional degradation in aging cancer survivors [50].

The inflammatory blood marker *CRP* decreased in both our groups without achieving significance in within or between comparisons. In general, physical activity and exercise are likely to lower circulating cytokine levels in cancer survivors [70]. For our results, it is important to consider that mean concentration at baseline was within the reference range, therefore no significant reductions were likely.

An increase in *body cell mass* was evident in both groups with no group differences (mean difference of 0.3 kg between IG and CG). The changes we documented in body cell mass, as an essential component of lean body mass, are in accordance with the data (mean difference 0.41 kg) in a recent meta-analysis [71]. The *BMI* was unchanged within each group and after comparing both—a finding that is in line with two meta-analyses involving prostate and colorectal cancer patients [72, 73] however not breast cancer patients [74]. Improvements in body composition via strength training interventions in cancer survivors are markedly low but potentially clinically meaningful in cancer survivors [71]*.*

The effectiveness of training interventions in patients depends on their participation and *adherence* rates [31]. In the present trial entailing automatic activity feedback and counseling, about 74% of the patients engaged in at least 1.5 training sessions per week or 75% of the prescribed minimum of two sessions per week during the home-based exercise training period (6 months); however, this rate is 71 to 90% according to adherence data in the literature, [75, 76]. The average of these patients fulfilled the current guideline recommendations of at least 150 min of physical activity per week [28]. Nevertheless, despite feedback information and high app usage rates, only 56% of the IG patients participated in at least 2 exercise sessions per week and achieved a mean of 172 active minutes per week (< 3 MET).

Eighteen serious *adverse events* (16 patients; hospitalizations or cancer progression) unrelated to the exercise intervention occurred during the study period with no difference in incidence between IG and CG. The number of serious adverse events we observed is considerably higher than those reported in meta-analyses [36, 37, 76] and reflects our patients’ post-surgery condition. Overall, there is little reliable information on safety, particularly the reporting of serious adverse events, in studies reporting on home-based training [76]. Overall, exercise is generally safe for cancer survivors [6, 8] and can be adapted by selecting indication-specific training parameters (range of joint motion, intensity, training position). However, after surgical cancer therapy or during chemotherapy, individual patient limitations and problems should be considered.

## Limitations

This study has several limitations. First, our CG patients, like those in the IG, underwent activity tracking including feedback information, which was probably a major motivating factor for a more active lifestyle [77, 78]. If we had blinded the CG study subjects, their risk of dropout from wearing an activity tracker for 6 months without feedback information would have been high. Second, the physicians and study nurses who conducted the examinations, as well as the patients, were also not blinded, which could have had an effect on results. Third, the SARS-CoV-2 pandemic, involving surgery cancelations, high patient refusal rates, and restrictions on public life, substantially reduced the attendance rates and precluded entity subgroup analysis. In addition, study funding was time-based, which prohibited us from enlarging our sample size after the funding period. The sample sizes per entity we had anticipated were not realizable. We therefore conducted a cross-entity evaluation. Fourth, the short intervention period does not permit the interpretation of long-term effects on recurrence and mortality. Fifth, we were unable to obtain impedance cardiography without artifacts in all patients. Sixth, this study most likely involved exercise-experienced patients considering the study focus on physical activity [31]. As most of our study parameters were consequently in the reference range, they were unlikely to improve.

## Conclusions

Home-based and body-weight online training among postoperative cancer patients yielded a difference in the V̇O_2max_ change at 6 months between those patients assigned to training compared to control patients given activity feedback, but the IG patients did not achieve the prespecified assumed difference in V̇O_2max_. The change in RPP in the IG confirms the change in V̇O_2max_. In addition, providing digital activity feedback seems to have an influence on motivating patients in the control group to practice a healthier lifestyle, similar to the intervention group. Home-based online training might be an effective component after cancer surgery and provides the opportunity for area-wide implementation into cancer care. These findings support large-scale studies investigating long-term effect of online-based training with different patient groups, training volumes, and intensities, as well as automated activity feedback provision for patients with malignancies.

### Supplementary Information

**Additional file 1: Table S1.** Primary end point separated by subgroups. **Table S2.** Data of 3-month visit. **Table S3.** Per protocol analysis.

## Data Availability

The datasets generated during the present study can be obtained from the corresponding author on reasonable request.

## Abbreviations

BCM: Body cell mass
BMI: Body mass index
CG: Control group
CI: Confidence interval
CO: Cardiac output
CR10 scale: Category-Ratio scale
CRBP-TS: ColoRectal, Breast, and Prostate Cancer—Telemonitoring and Self-management
CRP: C-reactive protein
diff: Difference
DRKS: Deutsches Register Klinischer Studien (German Clinical Trials Register)
ECOG: Eastern Cooperative Oncology Group
EORTC QoL-C30: European Organization for Research and Treatment of Cancer Quality of Life Questionnaire
GCP: Good clinical practice
HbA_1c_: Glycosylated hemoglobin
ICD: International Classification of Diseases
IG: Intervention group
LTE: Long-Term Evolution standard
METs: Metabolic equivalent of tasks
min: Minute
mo: Months
pre: Baseline
QoL: Quality of life
RPP: Rate pressure product
SAE: Serious adverse event
SARS-CoV-2: Severe acute respiratory syndrome coronavirus type 2
SD: Standard deviation
TNM: Tumor, Nodes, Metastases
V̇O_2max_: Maximum oxygen uptake
