# Supplementary material for: Effect of home-based online training and activity feedback on oxygen uptake in patients after surgical cancer therapy: a randomized controlled trial
Source: BMC Med. 2023 Aug 8;21:293. doi: 10.1186/s12916-023-03010-6 (PMC10408062; doi:10.1186/s12916-023-03010-6)
Supplement: Supplementary file 1 — Additional file 1: Table S1. Primary end point separated by subgroups. Table S2. Data of 3-month visit. Table S3. Per protocol analysis. [file 12916_2023_3010_MOESM1_ESM.docx]

**Additional file 1 - Supplement**

**Table S1** Primary end point after 6 months by entity (intent to treat analysis)

|  | Mean (SD) [sample size] | | |  | | | |  | Time effect^c^ | | Group effect^c^ | | Inter-action effect^c^ | |
| --- | --- | --- | --- | --- | --- | --- | --- | --- | --- | --- | --- | --- | --- | --- |
|  | **Intervention group**  **____________________** | | | **Control group**    **_____________________** | | | | Estimates^b^ of differences IG vs. CG (95% CI) | p | | p | | Group x Time  p | |
|  | **pre** | **6 mo** | **Diff^a^** | **pre** | **6 mo** | **Diff^a^** |  | |  |  | |  | |  |
| Breast-Ca  VO_2max_, ml/kg/min | 25.6 (5.9) [41] | 26.9 (6.1) [35] | **1.5***  (2.4)  [35] | 27.8 (7.1) [41] | 30.4 (7.2) [33] | **1.1*** (2.5) [33] | 0.43 (-0.7 to 1.5) | | **<0.001** | | 0.19 | | 0.66 | |
| Prostate-Ca  VO_2max_, ml/kg/min | 28.4 (6.1) [23] | 31.0 (6.3) [22] | **2.4***  (3.2)  [44] | 29.1 (4.5) [21] | 29.8 (7.1) [17] | 0.6 (4.2) [17] | 1.8 (-0.4 to 4.0) | | **0.01** | | 0.89 | | 0.17 | |
| Colorectal-Ca  VO_2max_, ml/kg/min | 24.1 (4.4) [10] | 25.6 (6.2) [6] | 1.6*  (3.0)  [6] | 26.8 (6.2) [9] | 25.5 (6.63) [8] | -1.2 (5.1) [8] | 3.0 (-0.8 to 6.1) | | 0.79 | | 0.73 | | 0.15 | |

Abbreviations: pre = baseline; mo = months; diff = difference; VO_2max_ = maximum oxygen uptake; ^a^sensitive analysis: results of the complete case analysis considering all available data; ^b^estimates of differences between group changes after 6 months; ^c^main effects of mixed-effects models; *significant difference (p<0.05; within groups);

**Table S2** Primary and secondary end points after 3 months

|  | Mean (SD) [sample size] | | |  | | | |  |
| --- | --- | --- | --- | --- | --- | --- | --- | --- |
|  | **Intervention group**  **___________________** | | | **Control group**    **___________________** | | | | Difference^b^ 3 months  IG vs. CG (95% CI) |
|  | **pre** | **3 mo** | **Diff^a^** | | **pre** | **3 mo** | **Diff^a^** |  |
| VO_2max_, ml/kg/min | 26.2 (6.0) [74] | 27.2 (6.3) [66] | **1.2***  (2.6)  [66] | | 28.1 (6.3) [71] | 29.3 (6.8) [59] | 0.51 (2.9) [59] | 0.63 (-0.40 to 1.66) |
| VO_2max_, ml/min | 2045 (454) [74] | 2146 (489) [66] | **94*** (214) [66] | | 2065 (501) [71] | 2120 (545) [59] | 39 (216) [59] | 24 (-60 to 108) |
| Peak power output, watt | 132 (33.3) [74] | 138 (35) [67] | **5.5*** (13) [67 | | 136 (34.2) [71] | 144 (37) [59] | **5.9***  (16) [59] | 4.5 (-1.3 to 10.3) |
| Rate-pressure product^c^ | 26468 (5539) [73] | 25954 (5421) [64] | -132 (3140) [64] | | 26707 (5094) [71] | 26165 (5533) [57] | -372 (2458) [57] | **-1330**^#^ (-2441 to -218) |
| Cardiac output max, l/min | 16.7 (3.3) [61] | 17.3 (3.3) [59] | 0.64 (2.3) [50] | | 17.7 (2.8) [59] | 17.8 (3.5) [51] | -0.02 (2.2) [43] | -0.02 (-0.9 to 0.9) |
| CRP, mg/l | 3.2 (7.6) [76] | 2.06 (2.4) [66] | -1.3 (8.2) [65] | | 3.37 (14.5) [71] | 1.50 (1.6) [60] | -2.06 (16) [60] | -0.4 (-1.2 to 0.5) |
| Body cell mass, kg | 30.1 (5.8) [74] | 31.0 (6.1) [68] | **0.7*** (1.7) [67] | | 29.9 (6.2) [69] | 30,2 (6.5) [58] | **0.5*** (1.4) [58] | 0.1 (-0.5 to 0.7) |
| BMI, kg/m^2^ | 26.6 (4.7) [74] | 26.9 (4.5) [68] | -0.01 (1.0) [67] | | 25.3 (4.4) [71] | 25.0 (4.3) [60] | 0.06 (0.7) [59] | -0.2 (-0.5 to 0.07) |
| EORTC QLQ-C30; global score | 62.4 (21.6) [68] | 66.2 (19.7) [67] | 3.9 (24.8) [62] | | 60.1 (22.9) [65] | 68.5 (22.2) [60] | **8.9*** (23.5) [57] | -0.2 (-11 to 11) |
| Activity per week^d^ (>3 MET), min | 145 (160) [74] | 128 (150) [67] | -30 (131) [66] | | 173 (152) [69] | 137 (125) [62] | **-38*** (102) [60] | 15.2 (-20 to 50) |
| App use per week^d^ numbers | 26.2 (7.7) [75] | 23.0 (9.1) [72] | **-3.1*** (7.5) [71] | | 21.2 (6.6) [71] | 17.9 (5.2) [66] | **-3.4*** (5.2) [66] | -0.9 (-3.4 to 1.6) |

Abbreviations: pre = baseline; mo = months; diff = difference; VO_2max_ = maximum oxygen uptake; MET = metabolic equivalent of tasks; BMI = Body mass index; EORTC QLQ = European Organisation for Research and Treatment of Cancer Quality of Life questionaire; ^a^sensitive analysis: results of the complete case analysis considering all available data; ^b^estimates of differences between group changes; ^c^for the same absolute power output; ^9d^pre = week 1 to week 8 and 6 mo = week 17 to week 25; *significant difference (p<0.05; within groups); ^#^significant difference (p<0.05, between groups)

**Table S3** Primary and secondary end points after 6 months (per protocol analysis)

|  | Mean (SD) [sample size] | | |  | | |  |  | Time effect^c^ | Group effect^c^ | Inter-action effect^c^ |
| --- | --- | --- | --- | --- | --- | --- | --- | --- | --- | --- | --- |
|  | **Intervention group**  **___________________** | | | **Control group**    **___________________** | | | Difference^b^ 3 months  IG vs. CG (95% CI) | Difference^b^ 6 months  IG vs. CG (95% CI) | p | p | Group x Time  p |
|  | **pre** | **6 mo** | **Diff^a^** | **pre** | **6 mo** | **Diff^a^** |  |  |  |  |  |
| VO_2max_, ml/kg/min | 26.5 (5.9) [46] | 28.0 (6.8) [44] | **1.4***  (3.2)  [44] | 28.1 (6.3) [71] | 29.5 (7.1) [58] | 0.65 (4.0) [58] | 0.34 (-0.8 to 1.5) | 0.81 (-0.3 to 1.9) | **0.006** | 0.34 | 0.37 |
| VO_2max_, ml/min | 2106 (486) [46] | 2203 (555) [44] | **100 (241) [44]** | 2065 (501) [71] | 2149 (556) [58] | 55 (276) [58] | 8 (-84 to 99) | 42 (-48 to 133) | **<0.003** | 0.49 | 0.60 |
| Peak power output, watt | 136 (35.5) [46] | 146 (41.5) [44] | **9.3*** (15.4) [44] | 136 (34.2) [71] | 145 (38.6) [58] | **6.4*** (18.7) [58] | 5.0 (-1.4 to 11) | 3.3 (-3.0 to 9.7) | **<0.001** | 0.87 | 0.30 |
| Rate-pressure product^d^ | 26851 (5347) [45] | 26265 (5931) [43] | -785 (2777) [43] | 26715 (5099) [71] | 26382 (4641) [58] | 94.2 (3249) [58] | -1038 (-2188 to 113) | -690 (-1799 to 419) | 0.54 | 0.75 | 0.19 |
| Cardiac output max, l/min | 16.9 (3.2) [38] | 17.6 (3.7) [38] | 0.29 (2.5) [32] | 17.7 (2.8) [59] | 17.9 (3.5) [51] | 0.17 (2.7) [43] | -0.34 (-1.4 to 0.7) | 0.32 (-0.7 to 1.3) | 0.58 | 0.44 | 0.40 |
| CRP, mg/l | 3.91 (9.66) [45] | 1.73 (1.85) [43] | -1.01 (5.10) [43] | 3.37 (14.5) [70] | 1.55 (1.60) [59] | -2.12 (16.1) [59] | -0.2 (-1.0 to 0.7) | -0.3 (-5.1 to 4.6) | 0.28 | 0.69 | 0.93 |
| Body cell mass, kg | 30.6 (6.0) [45] | 31.6 (6.8) [43] | **1.0*** (2.2) [43] | 29.9 (6.2) [69] | 30.5 (6.7) [58] | **0.7*** (1.7) [58] | 0.4 (-0.2 to 1.1) | 0.4 (-0.3 to 1.1) | **<0.001** | 0.62 | 0.38 |
| BMI, kg/m^2^ | 27.2 (4.8) [45] | 27.1 (4.8) [43] | -0.2 (1.2) [43] | 25.3 (4.4) [71] | 25.1 (4.1) [60] | 0.2 (0.9) [60] | -0.2 (-0.5 to 0.15) | -0.3 (-0.6 to 0.04) | 0.43 | **0.04** | 0.22 |
| EORTC QLQ-C30; global score | 61.5 (21.1) [42] | 69.5 (24.5) [35] | 9.1 (24.5) [33] | 60.1 (22.9) [65] | 69.3 (18.8) [54] | **13.1*** (31.5) [50] | 2.3 (-9 to 14) | -0.6 (-12 to 11) | **0.002** | 0.97 | 0.86 |
| Activity per week^e^ (>3 MET), min | 189 (179) [45] | 150 (160) [45] | -40 (151) [45] | 170 (152) [69] | 114 (111) [60] | **-49*** (116) [58] | 8.4 (-34 to 51) | 12.7 (-35 to 59) | **<0.001** | 0.36 | 0.86 |
| App use per week^e^ numbers | 28.2 (7.2) [45] | 24.8 (7.7) [45] | **-3.3*** (8.9) [45] | 21.2 (6.6) [71] | 16.6 (7.1) [62] | **-5.0*** (7.0) [62] | 0.6 (-3.1 to 2.0) | 1.4 (-1.1 to 3.9) | **<0.001** | **<0.001** | 0.27 |

Abbreviations: pre = baseline; mo = months; diff = difference; VO_2max_ = maximum oxygen uptake; MET = metabolic equivalent of tasks; BMI = Body mass index; EORTC QLQ = European Organisation for Research and Treatment of Cancer Quality of Life questionaire; ^a^sensitive analysis: results of the complete case analysis considering all available data; ^b^estimates of differences between group changes; ^c^main effects of mixed-effects models; ^d^for the same absolute power output; ^e^pre = week 1 to week 8 and 6 mo = week 17 to week 25; *significant difference (p<0.05; within groups); ^#^significant difference (p<0.05, between groups)
